# Four-Dimensional Adjustable Electroencephalography Cap for Solid–Gel Electrode

**DOI:** 10.3390/s25134037

**Published:** 2025-06-28

**Authors:** Junyi Zhang, Deyu Zhao, Yue Li, Gege Ming, Weihua Pei

**Affiliations:** 1 The School of Advanced Technology, Xi’an Jiaotong-Liverpool University, Suzhou 215000, China; Junyi.Zhang21@student.xjtlu.edu.cn (J.Z.); Yue.Li@xjtlu.edu.cn (Y.L.); 2 The EEG Techonology Co., Ltd., Suzhou 215100, China; 3 The Biomedical Engineering, Hebei University of Technology, Tianjin 300000, China; deyuhebut@163.com; 4 The Department of Biomedical Engineering, Tsinghua University, Haidian District, Beijing 100084, China; minggege@mail.tsinghua.edu.cn; 5 The Laboratory of Solid State Optoelectronics Information Technology, Institute of Semiconductors, Chinese Academy of Sciences, Beijing 100083, China; 6 The University of Chinese Academy of Sciences, Beijing 100049, China

**Keywords:** solid–gel electrode, semi-dry electrode, adjustable EEG cap, brain–computer interface

## Abstract

Currently, the electroencephalogram (EEG) cap is limited to a finite number of sizes based on head circumference, lacking the mechanical flexibility to accommodate the full range of skull dimensions. This reliance on head circumference data alone often results in a poor fit between the EEG cap and the user’s head shape. To address these limitations, we have developed a four-dimensional (4D) adjustable EEG cap. This cap features an adjustable mechanism that covers the entire cranial area in four dimensions, allowing it to fit the head shapes of nearly all adults. The system is compatible with 64 channels or lower electrode counts. We conducted a study with numerous volunteers to compare the performance characteristics of the 4D caps with the commercial (COML) caps in terms of contact pressure, preparation time, wearing impedance, and performance in brain–computer interface (BCI) applications. The 4D cap demonstrated the ability to adapt to various head shapes more quickly, reduce impedance during testing, and enhance measurement accuracy, signal-to-noise ratio (SNR), and comfort. These improvements suggest its potential for broader application in both laboratory settings and daily life.

## 1. Introduction

The extracranially collected EEG signals exhibit distinctive features typified by low frequency and diminished amplitude, rendering them prone to disruption by internal and external factors within the biological milieu [1]. This poses challenges for the acquisition of non-invasive EEG recordings. When installing or placing electrodes on the scalp, it is necessary to have a specific device pressing the electrode on the scalp to fix it and ensure good contact between the electrode and scalp to realize the stable acquisition of EEG signals [2]. The apparatus typically is a head-adapted EEG cap to fasten all electrodes onto the scalp securely [3]. EEG caps are commonly fabricated using fabric, elastic polymers, or silicone rubber [4]. These caps are available in various sizes to accommodate different user head shapes and circumferences, ensuring optimal electrode–head contact to acquire effective EEG signals.

For conventional wet electrode caps, each electrode is individually injected with the gel between the scalp and the electrode [5]. Due to variations in head shape among individuals, gaps between the electrodes and the scalp must be filled with conductive gel, requiring a certain level of technical expertise from the experimenters [6]. When applied correctly, the gel’s inherent viscosity provides excellent electrical contact between the scalp and the electrodes, even in the absence of direct contact, ensuring low-noise acquisition of EEG signals [7]. However, insufficient gel application can lead to suboptimal impedance at that electrode site, making it more susceptible to noise interference [8]. Conversely, using too much gel can cause short-circuiting [9]. Additionally, participants must wash their hair after the experiment, adding complexity to the procedure and potentially causing inconvenience [10]. Although wet electrode caps maintain good electrical conductivity, their use outside the laboratory is limited due to the cumbersome nature of the experimental process.

Dry electrode EEG caps are another type of EEG acquisition device, and they are known for their convenience of use [11]. Compared with traditional wet electrode EEG caps, dry electrode EEG caps do not require electrolytic gel or liquid to ensure good electrode contact, making them easier to wear and reducing the complexity of cleaning and maintenance [12]. For the user, it requires little maintenance. Through structures such as claw-style [13], pillar-style [14], comb-style [15], and brush-style [16], it makes direct contact with the scalp through the hair [17]. The pressure exerted on dry electrodes can significantly influence their impedance characteristics [18]. Proper pressure in both direction and magnitude is crucial for suitable contact impedance of dry electrodes [19]. Due to the limited elasticity of fabric caps, the specificity of the head shape directly affects the degree of contact between the scalp and the dry electrodes. Moreover, adjusting a single electrode often affects the contact status of surrounding electrodes. To address this issue, some institutions have changed the electrode structure, including Lin et al. [20], who have designed a dry-type active electrode that mainly focuses on the cylindrical dry electrode’s column by providing a scalable structure that can compensate for the irregularities of the skull itself. Following the same principle, Wu et al. [21] have designed a screw dry electrode, which adjusts the electrode height by adding a rotatable structure to the top of the cylindrical dry electrode. Liu et al. [22] developed a new retractable dry electrode device and applied it to the EEG cap.

Like the dry electrode, the Preset Gelled (PreGel) electrode also needs to pay attention to the problem of electrode contact with the scalp [23]. The PreGel electrode is a novel form of the semi-dry electrode; it uses cross-linked hydrogel, which can trap much water without easy loss. Compared with wet electrodes, the PreGel electrode in an EEG cap offers better convenience, and compared with dry electrodes, it has lower contact impedance, making it highly convenient for long-term EEG recording in conjunction with the EEG cap [20]. However, the part of the PreGel electrode that comes into contact with the skin has a specific preformed shape. Suppose the PreGel is not pressed on the scalp and the hair is moistened; there will be air gaps or spaces between the PreGel (this kind of gel is solid). In that case, the conductive pathway between the scalp and the electrode will be significantly affected or blocked, resulting in a significant decrease in the SNR of the brainwave signal. Therefore, compared with the wet electrode cap, the dry or semi-dry electrode cap needs better adaptability and adjustment ability for the head circumference and shape.

The ordinary EEG cap only features tightening structures on both sides of the chin [24]. As a result, when the cap is tightened on both sides, the electrodes on the top of the head are pressed tightly by the cap, ensuring good contact with the scalp. Typically, there is a large angle between the temporal and cranial areas [25]. When the cap is pulled from both sides of the chin, the pressure adjustment on the temporal electrodes is quite limited. For the electrodes in the occipital area, the orientation of the occipital region and the top of the skull is almost perpendicular, resulting in a more challenging state of compression for the electrodes in this area. In contrast, when tightening the cap at the chin area, the electrodes are not under compression but rather, to some extent, relaxed. In other words, due to the deviation of the skull shape from the standard spherical shape, it is challenging to ensure that the cap is tightened in every part of the head when there is only one dimension for tightening the EEG cap. By making the electroencephalography caps more adjustable, we can ensure that most of them can be tightened to compress all the electrodes on the head. However, based on current research findings, the improvements made to the EEG cap itself predominantly utilize rigid materials. Takumi et al. [26] have developed a helmet straw hat with a “shutter” device mounted on top, equipped with dry electrodes to part the hair and enhance contact. Lin et al. [27] and Chi et al. [28] independently developed dry-electrode EEG devices composed of multiple rod-shaped rigid resin strips. These modular designs enable free reconfiguration, offering a structural solution to the fitting limitations inherent in conventional EEG caps. Kim et al. [29] have developed a rigid eight-channel comb-shaped dry electrode headband, enabling rapid measurement of brain electrical signals. Due to the low flexibility of this hard-wearing headgear, it often can only accommodate a small number of electrodes in practical applications, thus being unable to achieve full-brain compatibility. Upon this foundation, we developed a highly adaptable 64-channel 4D adjustable EEG cap.

By utilizing the unique characteristics of the human skull, we have developed a 4D adjustable EEG cap made from materials like silicone elastomer and elastic fabric, allowing it to conform to adult head shapes. The cap can be adjusted in four dimensions, facilitating a better fit for various head shapes. Its structural advantages enable multiple electrodes on the EEG cap to be uniformly adjusted simultaneously, ensuring comfortable wear and low contact impedance. In BCI applications, the 4D cap demonstrates superior performance compared with non-adjustable EEG caps in the Steady State Visual Evoked Potential (SSVEP) paradigm.

## 2. Materials and Methods

### 2.1. Cap and Electrode

The 4D adjustable EEG cap is designed to fit the varied shapes of adult skulls. This study uses statistical data on head length, width, and circumference from the Chinese National Ergonomic Survey (CNES), conducted from 2014 to 2018 [30]. By analyzing the average measurements from this dataset, we developed a 3D head model using SOLIDWORKS. Based on this model size, we crafted an EEG cap and designed the 4D adjustment feature to accommodate the differences in human head shapes.

The first dimension Length (L) is the horizontal circumference of the head. The second dimension Height (H) is the central axis length from brow to occiput. The third dimension Side (S) pertains to the curvature adjustment of the occipital region, and the fourth dimension Front (F) can be adjusted along the temporal-parietal length, spanning from the ear to the chin. All dimensions—L, H, S, and F—can be adjusted independently, enabling the cap to fit various head shapes by altering its size and form in these four directions.

**Figure 1:**
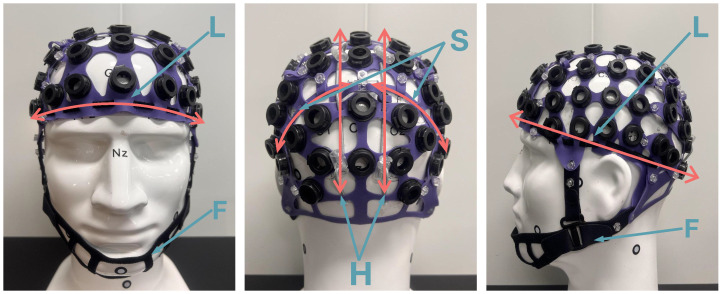
Pink arrows indicate the 4D adjustable EEG cap features four adjustment directions: L (Length), H (Height), S (Side), and F (Front).

In line with this adjustment design, we utilized a silica gel sheet as the body of the 4D cap, which offers excellent elasticity and flexibility with a thickness of 1.5 mm. The 4D cap with optimized mechanical properties for cranial adaptation: Young’s modulus of 0.5–1.2 MPa (matching scalp tissue compliance) and elongation at break of ~140%. As shown in Figure 1, the 4D control device consists of the following:

(1) L dimension: This dimension features a non-elastic flat band that threads through the cap, encircling the head’s horizontal circumference. It can be adjusted to fit various head sizes.
(2) H dimension: This involves an adjustable drawstring for the anterior-posterior diameter located in the occipital region at the back of the head. It allows for changes in the central axis length from brow to occiput. The drawstring extends from the mid-forehead to the occipital protuberance line, parallel to the sagittal suture. Two symmetrically positioned drawstrings on the left and right sides facilitate size adjustments in the occipital area.
(3) S dimension: this dimension employs the same silicone material as the cap body, enabling adjustment of the occipital region to the horizontal circumference at an angle of approximately 45 degrees.
(4) F dimension: this dimension utilizes fabric with Velcro, consistent with the adjustment mechanism of a traditional EEG cap.

By adjusting the length or the tension of the rope and simultaneously modifying the spacing of the electrode lines threaded through it, uniform distribution of electrode spacing is maintained as dimensions change. This approach prevents concentration of adjustments on just a few electrodes. The cap includes multiple hollow areas to facilitate electrode positioning and enhance contact between the PreGel electrodes and the scalp. With its adjustable design, each electrode on the cap can closely adhere to the scalp. The adjustable ranges of the 4D cap are detailed in Table 1.

To assess the usability of the 4D cap adjustable range, a study involving 30 adults (with an equal gender ratio) was conducted to gather data on head characteristics across four dimensions. Table 2 outlines the measurements for the L, H, S, and F dimensions. The findings indicate that all measurements fall within the adjustable range of the 4D cap. After making the necessary adjustments, the cap was found to be suitable for all participants.

The 4D cap follows the 10–20 standard lead placement system, featuring assembly positioning holes that are labeled with the corresponding codes for each electrode. To ensure a secure fit, the dimensions of these positioning holes are designed to be slightly smaller than the electrode slots, facilitating proper assembly and stability during use.

**Figure 2:**
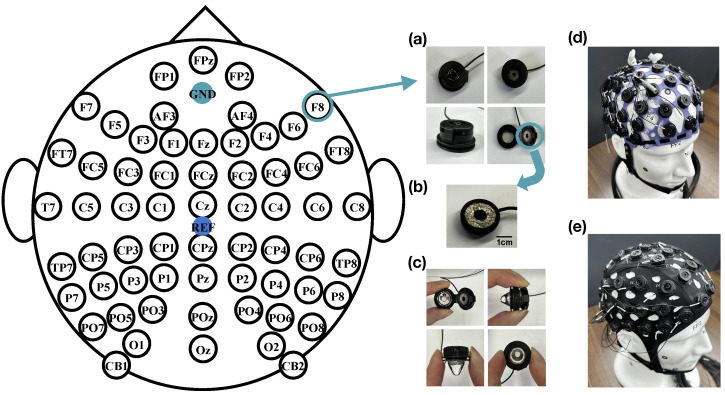
Equidistant electrode layout with 64 channels; (**a**) the electrode placement device after redesign; (**b**) the powder-sintered Ag/AgCl electrode; (**c**) the electrode device after assembling the PreGel; (**d**) the 4D adjustable EEG cap; (**e**) the COML non-adjustable EEG cap.

The reference electrode is placed at the CPz position, and the ground electrode is placed at the GND position, as shown in Figure 2. The electrodes of the 4D cap are based on our previous development of PreGel electrodes, suitable for areas with or without hair, and with a correlation coefficient of over 95% with wet electrode EEG signals [23]. In addition, we have redesigned the electrode placement device to be more convenient than the previous version, significantly reducing the preparation time for experiments. As shown in Figure 2a, the electrodes are mainly composed of hydrogel probes and sintered silver chloride powder. The snap-on cover and house of a PU-based material are used to assemble the hydrogel probe and Ag/AgCl electrode. In Figure 2b, a powder-sintered Ag/AgCl electrode with a diameter of 8 mm was placed onto the lid to provide optimal electrochemical characteristics. The hydrogel probe is placed inside a hollow circular shell with a diameter of 7 mm, and after filling the hydrogel, it can be directly fastened together by a snap. In Figure 2c, this device allows for the repeated opening and closing for the replacement of the hydrogel. The design of the hydrogel electrode probe is conical, with a pointed tip for better penetration of the hair and close contact with the scalp and a flat end for complete contact with the powdered sintered Ag/AgCl electrode on the snap-on cap, ensuring the rate of ion transmission and high conductivity of the entire device. The lower end of the shell is provided with a circular groove, allowing the electrode device to be mounted on the assembly hole reserved in the EEG cap. The electrode lead is directly connected to the Ag/AgCl electrode, with the length determined by the characteristics of the wireless or wired amplifier, typically selected to be between 10 and 150 cm. The physical picture of the 4D cap is shown in Figure 2d.

As illustrated in Figure 2e, we utilized a COML non-adjustable EEG cap (GREENTEK), with the PreGel electrodes and the arrangement the same as that of the 4D adjustable EEG cap, as an object of comparative testing to evaluate the proposed 4D cap.

### 2.2. In Vivo Test

The in vivo tests compare and evaluate the 4D cap with the COML cap in four aspects. This encompasses contact pressure, preparation time, skin–electrode impedance, and SSVEP-BCI performance.

To minimize potential deviations in results due to tester actions, this experiment was conducted by the same operator. Before using the 4D cap, it is crucial to cleanse the scalp areas where the reference and ground electrodes will be placed using cotton pads soaked in ethanol. Other electrode sites do not require specific skin cleansing.

The operator should optimize contact between the electrodes and the scalp in individual channels with less-than-ideal impedance performance. Impedance should be adjusted one channel at a time, following a front-to-back and left-to-right sequence. A channel is considered properly adjusted when the impedance is below 50 KΩ [23]. Additionally, the adjustment time for each experiment is limited to 20 min.

The volunteers were equally distributed by gender (1:1) and had an average age of 25 ± 5 years. A preliminary assessment of their mental, psychological, and medical conditions confirmed that all participants were healthy and not taking any medication. Volunteers were instructed to use pH-neutral shampoo the night before the study and to ensure they had at least 7 h of sleep. The 4D cap and the COML cap were evaluated with a minimum interval of 12 h between tests. This recovery period was implemented to minimize the effects of hydration from the electrolyte solution seeping out of the PreGel electrodes on the scalp.

Volunteers’ subjective comfort and attention levels were assessed before and after EEG recording using the Scott and Huskisson pain scale, which ranges from 1 to 10 [31]. All experiments in this study received approval from the Ethics Committee of Tsinghua University and were conducted in accordance with relevant guidelines and regulations. Written informed consent was obtained from all volunteers prior to their participation.

The data acquisition for the EEG and impedance measurements was performed on a Neuroscan EEG acquisition system (Synamps2) using the Curry 8 software. All data was exported in raw format and analyzed using custom MATLAB 2023 scripts (The MathWorks, Natick). The Neuroscan 64-channel amplifier features an input impedance of >1 GΩ, a common mode rejection ratio of >100 dB, and supports active shielding. It operates at a sampling rate of 1024 samples per second per channel.

### 2.3. Contact Pressure

To systematically evaluate the pressure–impedance relationship of hydrogel electrodes, we use MEMS-based force monitoring (MEMSensing) (100 Hz sampling rate) for precise contact pressure control (0.1–2.1 N range) synchronized with wideband impedance spectroscopy (5–128 k(Hz); DATA ACQUISITION/MULTIMETER SYSTEM). Each electrode configuration underwent 10 repeated measurements under controlled environmental conditions (23 ± 1 °C, 45 ± 5% RH), with ensemble averaging applied to derive the fundamental pressure–impedance characteristics.

For comparative assessment between EEG cap systems, we employed an ISO 10993-10 [32] compliant anthropomorphic head phantom featuring realistic scalp elasticity (Shore A 20–25), anatomical curvature variations (radius: 60–140 mm), and standardized hair density distribution. The experimental design included (1) the 4D adjustable cap and a commercial COML reference cap; (2) twelve representative electrodes (three per lobe: frontal [Fp1, Fpz, Fp2], temporal [C5, Cz, C6], parietal [P3, Pz, P4], and occipital [O1, Oz, O2]) to ensure coverage of major functional regions; and (3) after all the electrodes were fully assembled, the impedance values of the 12 electrodes on the two caps were recorded respectively. The impedance value is put into the pressure impedance curve to find the corresponding pressure value and compare the contact degree between the two hats and the scalp. Each point position was recorded 10 times and finally averaged to eliminate errors.

### 2.4. Preparation Time

The preparation time for the EEG cap in this study is divided into four parts: the gel probe installation, wear, impedance adjustment, and maintenance. For the 4D cap, the time taken to install 64 disposable gel probe electrodes on the electrode array is recorded as the installation time. The time taken to place the cap on the volunteer’s head and adjust it for a perfect fit using a 4D adjustment device was recorded as the wear time. Using tools such as cotton swabs through the assembly side apertures, the time taken to separate the hair between the electrode contact points and the scalp and adjust the impedance between the two interfaces to below critical value is recorded as the impedance adjustment time. All the gel probes were removed at the maintenance time.

The COML non-adjustable EEG cap in the same manner to record the preparation time.

### 2.5. Impedance Test

The impedance of the 62-channel electrodes was recorded ten times for each volunteer. The initial impedance test was conducted prior to the start of the SSVEP experiment. Continuous recordings were made throughout the experiment, and the final recording took place after the SSVEP experiment concluded.

A channel was considered properly adjusted when the impedance was below 50 kΩ, following manufacturer specifications for our acquisition system and accounting for the baseline impedance characteristic of solid–gel electrodes. The relative channel reliability (*CR*) is defined according to Equation (1), where *R* represents the number of channels marked as reliable channels in the cap, and *C* represents the total number of channels.

(1) $$CR=\frac{R}{C}\times 100\%$$

### 2.6. Performance in SSVEP-BCI System

A single-target experiment was conducted to compare the SSVEP responses collected using two types of PreGel EEG caps. A square flickering stimulus (200 × 200 pixels) with a stimulation frequency of 6 Hz was displayed at the center of a 24.5-inch liquid crystal display (LCD) with a resolution of 1920 × 1080 pixels and a refresh rate of 60 Hz. The experiment included 20 trials, each consisting of a 4 s stimulation period followed by a 1 s rest. The stimulation programs were developed using Psychophysics Toolbox Version 3 (PTB-3) in MATLAB [33]. Five volunteers participated in this experiment. They were seated upright in a chair, positioned 70 cm from the display, in a brightly lit room with controlled brightness conditions. A headrest was used to support their heads, minimizing movement and reducing artifacts in the EEG data. The EEG device digitized data at a sampling rate of 1000 Hz with 24-bit A/D conversion. For preprocessing, high-pass (0.1 Hz) and low-pass (100 Hz) zero-phase filters were applied, along with a 50 Hz notch filter to remove electrical noise.

Firstly, the amplitude and SNR of the SSVEPs recorded from the single-target experiment were analyzed using 4 s data epochs. The amplitude spectrum y(f) was computed using the Fast Fourier Transform (FFT). In accordance with Equation (2), the SNR in units of decibels (dB) was defined as the ratio of the amplitude y(f) at the stimulation frequency *f* to the mean amplitude of the sidebands (i.e., four adjacent frequencies with a 0.25 Hz interval on each side):

(2) $$SNR=20\log_{10}\frac{8\times y(f)}{\sum_{k=1}^{4}\left[y\left(f-0.25\times k\right)+y\left(f+0.25\times k\right)\right]}$$

Subsequently, the SSVEP intensity recorded with different caps was further compared by evaluating the classification performance of the phase-coded data segments. The SSVEP response maintains a strong phase-locking characteristic with the stimulus signal. By introducing a time shift to the SSVEP data segments based on the stimulation frequency, their phase information can be altered. The 4 s data were used to extract four 0.9 s long phase-coded SSVEP epochs corresponding to the four phases (0, $\frac{\pi }{2}$, π, $\frac{3\pi }{2}$). The task-related component analysis (TRCA) algorithm is utilized to detect different phases, employing bandpass filtering to extract the effective sub-band components of SSVEP, with a filtering range of 4–90 Hz [34]. Classification accuracy is a standard metric for evaluating the performance of BCI systems, defined as the ratio between the number of correctly identified targets in the target selection task and the total number of targets.

## 3. Result

### 3.1. Contact Pressure

**Figure 3:**
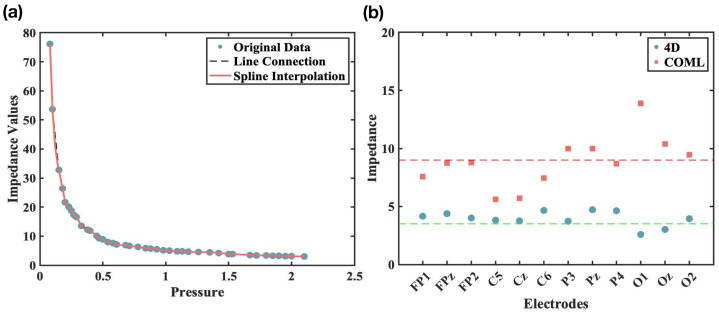
(**a**) The relationship between applied pressure and contact impedance for the PreGel electrode. (**b**) Impedance performance of the two EEG caps under different channels.

In Figure 3a, the nonlinear relationship between applied pressure and contact impedance is characterized for the PreGel electrode. The study identified an optimal pressure range of 0.2–2.1 N for stable electrode–model interface impedance (3–25 kΩ). The pressure–impedance correlation curve exhibited significant impedance fluctuations (>30 kΩ) at suboptimal pressures (<0.2 N). But on the two types of EEG caps, the PreGel electrode shows different contact effects. The 4D adjustable cap demonstrated superior performance compared with the COML reference. Figure 3b shows that under the same pressure conditions, the impedance of the COML cap is higher than that of the 4D cap, which indicates that the COML cap requires a higher pressure to achieve comparable impedance values. This result quantitatively validates the efficacy of the 4D system’s pressure regulation mechanism.

### 3.2. Preparation Time

The results indicate significant differences in preparation time between the 4D adjustable EEG cap and the COML non-adjustable EEG cap. The physical structure of the EEG cap is the primary factor influencing variations in preparation time.

**Figure 4:**
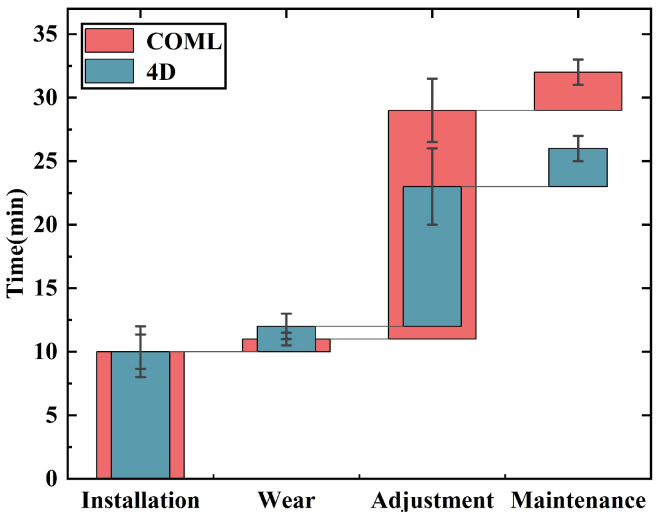
The preparation time for the two types of EEG cap.

As shown in Figure 4, the impedance adjustment time accounts for the majority of the difference in preparation time between the two caps. The 4D cap requires approximately 3 min to adjust for different head shapes during the wearing process. Compared with the direct wearing of the COML cap, this process involves several additional adjustment steps. However, these steps are crucial for the subsequent impedance adjustment. The average impedance adjustment time is 11 ± 3.2 min for the 4D cap, compared with 17 ± 2.45 min for the COML cap. The fit of the cap to the head shape directly influences the electrode–skin contact, with a better fit leading to faster impedance adjustment. Additionally, the 4D cap provides operators with enhanced flexibility for localized adjustments. For instance, if an electrode in the occipital region requires tightening, the operator can easily adjust the cap by tightening the drawstring in the H dimension, minimizing the impact on other electrodes. This feature makes the 4D cap more user-friendly than the COML cap, resulting in a quicker setup. As a result, the 4D cap enables the experimental process to be completed in less time.

### 3.3. In Vivo Impedance

**Figure 5:**
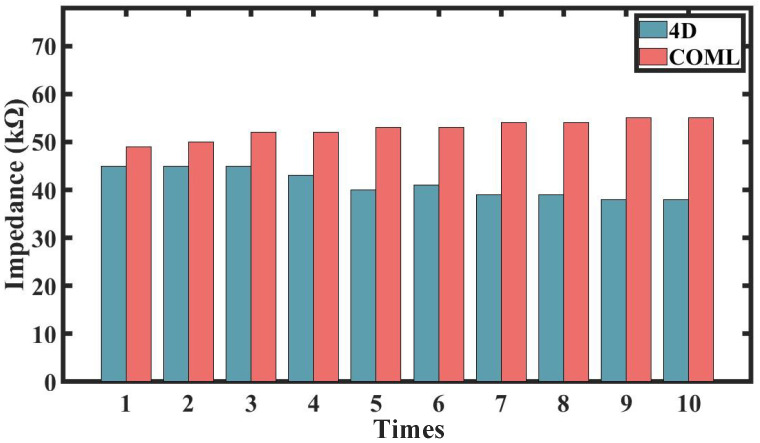
Electrode–skin impedance from the beginning to end of the two cap experiments, with 10 records.

Figure 5 shows the average of 10 recordings of the skin–electrode impedance from the beginning to the end of the experiment. For the COML cap, the average electrode–skin impedance across all channels was 49 KΩ at the start and increased to 55 KΩ by the end, indicating an upward trend. In contrast, the 4D cap started with an average impedance of 45 KΩ and decreased to 38 KΩ by the end, reflecting a downward trend.

The closer fit of the 4D cap to the head shape, combined with its adjustment device, ensures more secure contact between the electrodes and the skin, reducing the likelihood of displacement or loosening. As a result, the 4D cap maintains consistent pressure on the PreGel electrodes, allowing the hair and scalp to be gradually and fully humidified, leading to a steady decline in impedance. In contrast, the COML cap is more likely to loosen or shift due to insufficient contact pressure, which can cause a slight increase in impedance.

**Figure 6:**
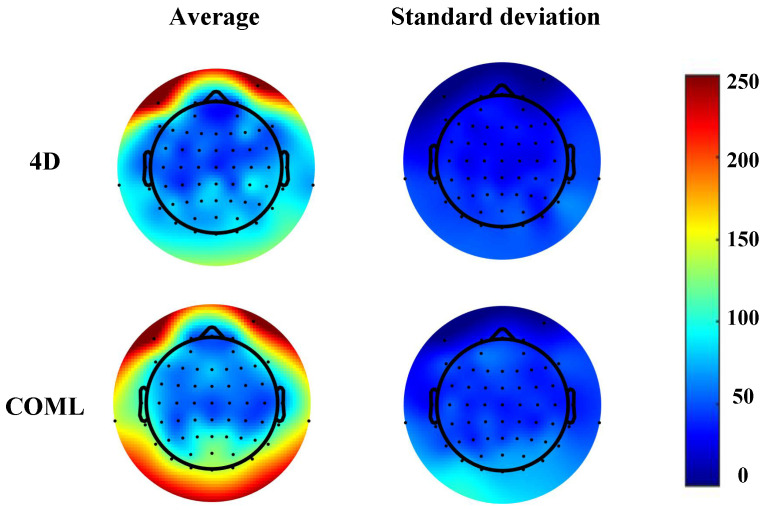
Topographic distribution of two caps with average and standard deviation of impedance for all subjects.

To further demonstrate the performance of the 4D cap, we compared the results obtained from the two caps, as shown in Figure 6. Both caps exhibited lower impedance in the frontal, prefrontal, and temporal regions, with the frontal region particularly showing the lowest impedance. This can be attributed to the lack of hair in the frontal scalp area, which allows the electrodes to make direct and rapid contact with the skin, thereby reducing the impedance at these sites. The prefrontal and temporal regions also exhibited lower mean and standard deviation values, suggesting consistent and reliable electrode contact in these areas. Conversely, higher impedance values were observed in the central, parietal, and occipital regions, particularly with the COML cap. The standard deviation of impedance in these regions was relatively higher, indicating less consistent contact in areas with more hair, which is typical for most participants. This can affect the quality of EEG data due to the variation in electrode–skin contact. The 4D adjustable EEG cap exhibited lower impedance and more uniformity in these regions, likely due to its adjustable design that allows for a more secure and consistent fit across different scalp areas. This improved fit contributes to better signal acquisition in regions with more hair, where impedance is generally higher.

**Figure 7:**
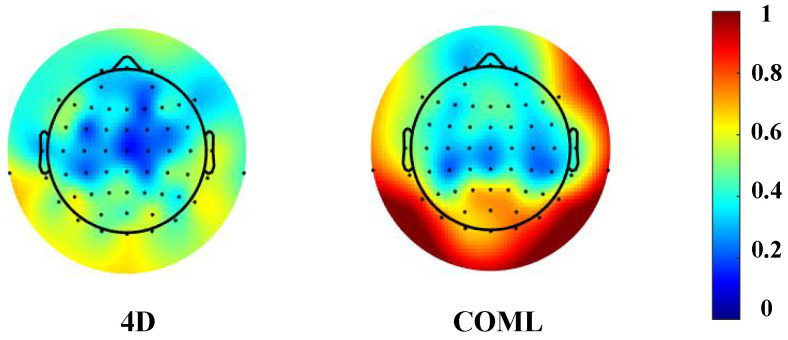
Topographic distribution of the relative channel reliability of the 4D caps, calculated based on the relative CR for all subjects.

According to the determination of the critical values for each channel, we calculated the relative CR for these two caps of the impedance recordings. As shown in Figure 7, the final result is an average channel reliability of 89% for the COML cap and 97% for the 4D cap. The region where the impedance decreases is consistent with the increase in electrode reliability. The adjustment device of the 4D cap has improved the impedance of the entire brain area, with a particularly noticeable improvement in the occipital region.

### 3.4. Performance in SSVEP-BCI System

**Figure 8:**
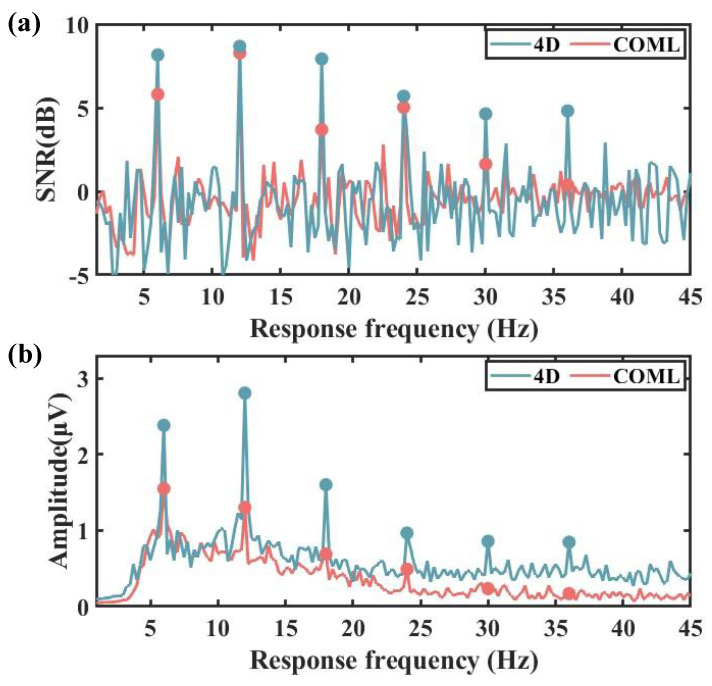
(**a**) The mean SNR of all subjects with the 4D adjustable EEG cap and the COML non-adjustable EEG cap. (**b**) The mean FFT of all subjects with the 4D adjustable EEG cap and the COML non-adjustable EEG cap.

Figure 8a shows the average amplitude spectrum and SNR of the SSVEP. Obvious peaks are evident at the fundamental frequency, along with substantial amplitudes at the second and third harmonics. At 6 Hz, the average amplitude spectrum peaks for the COML cap and the 4D cap are 1.4 μV and 2.4 μV, respectively, with the 4D cap demonstrating lower noise and higher signal quality.

The amplitude values from the 64 channels were mapped onto a brain area topography. As shown in Figure 8b, the results of the FFT indicated clear brain electrical signals at specific frequencies, with the most pronounced activity occurring in the occipital region. The COML cap showed an average value above 1.2 μV, while the 4D cap averaged above 2.1 μV. A *t*-test was conducted on the values from each channel of the two types of caps, revealing a significant difference (*p* < 0.05).

**Figure 9:**
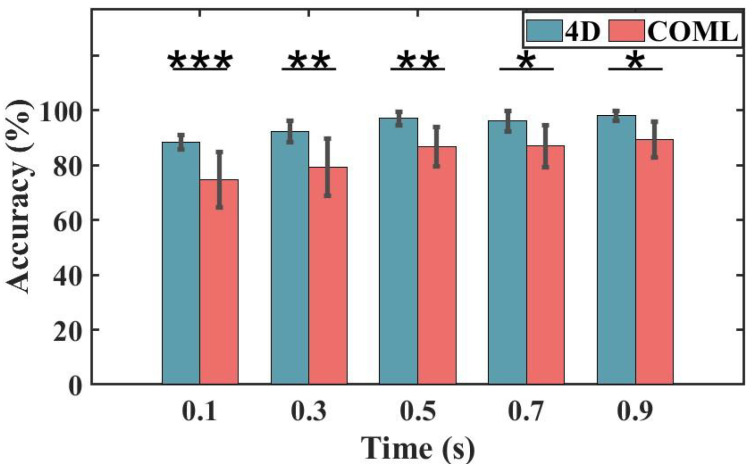
The average offline accuracy of the two caps at different data lengths by the TRCA algorithm. Error bars indicate one standard deviation. The asterisks indicate a difference in the pairwise comparison results (* *p* < 0.05, ** *p* < 0.01, and *** *p* < 0.001).

As shown in Figure 9, the two types of caps exhibit significant individual differences in classification accuracy, with the 4D cap demonstrating superior signal transmission performance. For the COML cap, the average accuracy among all participants was 93.33%, and the 4D cap achieved an average accuracy of 98%. A *t*-test indicated a significant difference (*p* < 0.05) in classification accuracy between the two caps.

The study included a questionnaire to gather experiential feedback from the volunteers. After the experiment, each volunteer was asked to complete the questionnaire. They reported no unpleasant skin sensations or pain from either cap. Additionally, the 4D cap demonstrated a better fit and greater comfort. Most volunteers indicated a preference for the 4D adjustable EEG cap.

## 4. Discussion

We have developed a novel 4D adjustable EEG cap that accommodates the head shapes of nearly all adults. Previous research has also explored adjustable caps; for instance, Chi et al. [35] designed a 32-channel headband utilizing soft fabric integrated with dry electrodes, which can adapt to various head shapes through horizontal and vertical adjustments via multiple straps. This flexible design has provided significant inspiration and insights for our work. Building on this foundation, our 64-channel EEG cap not only doubles the number of channels but also enhances the modulation dimensions, providing improved adaptability and performance. Most notably, while our system achieves 50–100% lower occipital impedance than Chi et al.’s 32-channel headband [35], we acknowledge that their design exhibits greater flexibility in rapid donning/doffing scenarios due to its simplified strap-based adjustment mechanism. This trade-off between comprehensive adaptability (our design) and operational speed (Chi et al.’s approach) represents an important consideration for different application contexts. On this basis, we also compare the research results of Takumi et al. [26], as detailed in Table 3. Compared with previous similar work, the 4D cap is more promising and user-friendly for BCI applications in terms of impedance, material, contact force, and stretchability.

The 4D cap reduces the preparation time required by 20% compared with the COML cap. Additionally, the adjustment device of the 4D cap allows for better fitting of the electrodes to the skin, resulting in improved impedance performance. To further our research, we conducted additional experiments comparing commercially available wet electrode EEG caps with dry electrode EEG caps as the additional control groups.

**Figure 10:**
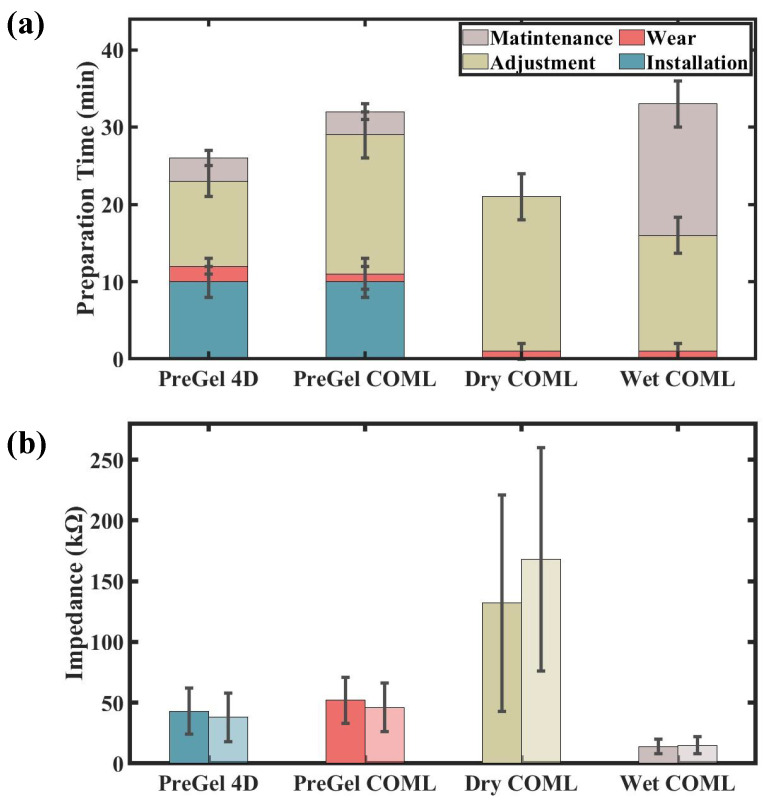
(**a**) Preparation time for four types of EEG cap. Error bars indicate one standard deviation. (**b**) Electrode–skin impedance maps at the beginning and end of the four caps experiments, with dark colors indicating before the experiment and light colors indicating after the experiment.

As shown in Figure 10a, the dry electrode cap requires no time for installation or maintenance. While the 4D EEG cap performs admirably, it is still less convenient and efficient compared with the dry electrode EEG cap. However, the dry electrode EEG cap requires more effort during the impedance adjustment step. Since there is no electrolyte to wet the skin in dry contact, minimizing interference at the skin interface is essential. To achieve the desired impedance for optimal contact, a secondary local scalp cleaning of individual electrodes is required [36]. This step significantly increases the adjustment time. In contrast, both the wet cap and the PreGel cap do not require this additional step. For wet electrode EEG caps, significant time is required for maintenance. After use, the cleaning process is essential—not only to rinse off the residual gel with water but also to wait for the cap to dry before it can be used again. In contrast, the PreGel and dry electrode EEG caps only require electrode removal, making them quicker and easier to maintain.

Correspondingly, we analyzed the impedance results presented in Figure 10b. Under consistent experimental conditions, the wet electrode EEG cap exhibited the best performance, with an average electrode–skin impedance of 15 KΩ.

**Figure 11:**
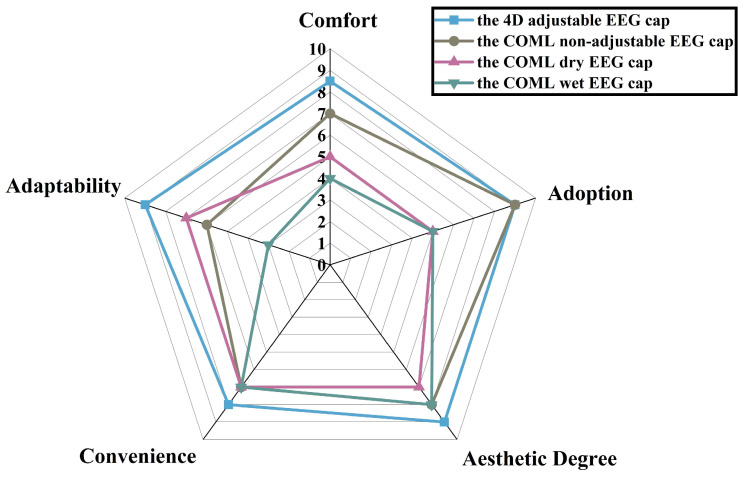
The average score of the volunteers on the five feelings questionnaire.

As shown in Figure 11, there is feedback from multiple volunteers. The average comfort rating for the 4D adjustable EEG cap is approximately 8, compared with around 6 for the COML non-adjustable EEG cap. In contrast, the COML dry electrode EEG cap consistently receives a rating below 5, while the COML wet electrode EEG cap averages around 5. This indicates that the gel electrode cap provides a higher level of comfort than both the dry and wet electrode caps. Most volunteers expressed a preference for the 4D adjustable EEG cap, likely due to the cumbersome adjustment process associated with the COML cap. In addition, the 4D cap is more consistent with the head shape and will reduce discomfort and pain during the experiment.

The integration of PreGel electrodes with our multidimensional adjustment framework represents a substantial methodological advancement: (1) preparation time is reduced by 15% compared with wet electrode caps, (2) impedance results metrics surpass dry electrode caps and (3) maintaining exceptional user comfort. This tripartite optimization is particularly significant for EEG applications.

Several study limitations must be acknowledged to properly contextualize these findings. While the 4D adjustable EEG cap demonstrates significant advantages for standard adult populations, several design limitations warrant discussion. First, the current adjustment ranges (head circumference: 52–62 cm; cephalic index: 75.2–85.6), while covering 89% of Chinese adults in our CNES-based validation cohort (n = 30), may not be particularly accurate across age extremes—particularly in groups of children where head circumferences below 50 cm are common or in individuals with pronounced brachycephaly/dolichocephaly (cephalic index < 74 or >86). Second, the occipital curvature adjustment shows empty for skull deformities (>10% deviation from normative curvature). These limitations reflect necessary trade-offs between universal adaptability and mechanical complexity. Our 30-subject validation included head circumferences spanning the 5th–95th percentiles (53–60 cm) and cephalic indices from 75.2 (mild dolichocephaly) to 85.6 (mild brachycephaly). Explicitly state these represent 89% coverage of the adult Chinese population per CNES. Addressing these constraints, we plan to develop (1) a children-specific variant with scaled-down dimensions (targeting 45–55 cm circumference) and softer silicone materials and (2) extended-range occipital modules with ±15% additional adjustability. These iterations aim to expand the technology’s applicability while maintaining its core advantages in signal quality and user comfort.

## 5. Conclusions

The 4D adjustable EEG cap represents a significant advancement over traditional EEG caps, providing enhanced adaptability, comfort, and performance in BCI applications. Its locally adjustable design accommodates a broader range of head shapes, making it both convenient and quick to use. The consequent decrease in the average impedance of all electrodes is crucial for the growing number of dry or semi-dry electrode devices. This innovative EEG cap promises more convenient and efficient EEG data collection, both in the laboratory and in daily life.

## Figures and Tables

**Table 1:** The adjustment range of the 4D cap.

Dimension	Minimum (cm)	Median (cm)	Maximum (cm)
Length	52	57	62
Height	35	40	45
Side	20	23	26
Front	22	26	30

**Table 2:** Measurement data of 30 volunteers in 4D of the Length, Height, Side, Front.

Number	Gender	Length	Height	Side	Front
1	Male	57 cm	37 cm	21 cm	26.5 cm
2	Male	57 cm	38 cm	23 cm	25.5 cm
3	Female	55 cm	36 cm	23 cm	26.8 cm
4	Male	60 cm	37 cm	23 cm	29.5 cm
5	Female	57 cm	39 cm	22 cm	28.5 cm
6	Female	57 cm	37 cm	25 cm	28 cm
7	Male	60 cm	37 cm	20.5 cm	27.7 cm
8	Male	57 cm	36 cm	21.5 cm	27.5 cm
9	Female	55 cm	37 cm	23 cm	26.5 cm
10	Male	58 cm	38 cm	22 cm	27 cm
11	Male	58 cm	38.5 cm	22 cm	29 cm
12	Male	57 cm	37 cm	22 cm	28.5 cm
13	Male	56 cm	36 cm	22 cm	26.5 cm
14	Female	56 cm	38 cm	21.5 cm	23.5 cm
15	Female	54 cm	37 cm	23 cm	23.5 cm
16	Female	54 cm	35 cm	22 cm	25 cm
17	Female	54 cm	35 cm	21 cm	27 cm
18	Female	56 cm	38 cm	23 cm	26 cm
19	Male	60 cm	39 cm	23 cm	28 cm
20	Female	57 cm	38 cm	20 cm	24 cm
21	Female	56 cm	38 cm	21 cm	25 cm
22	Female	56 cm	38 cm	22 cm	27.5 cm
23	Female	54 cm	36 cm	23 cm	26.5 cm
24	Male	56 cm	36 cm	21 cm	28 cm
25	Male	59 cm	42 cm	22 cm	28.5 cm
26	Male	58 cm	39 cm	21.5 cm	27.5 cm
27	Male	57 cm	38 cm	23 cm	28 cm
28	Male	57 cm	37 cm	22 cm	27.7 cm
29	Female	53 cm	36 cm	22 cm	24 cm
30	Female	54 cm	35 cm	21 cm	23.5 cm

**Table 3:** Compared with previous similar work (Adjustable EEG CAP).

Type	Electrode	Impedance	Material	Stretchable	Pressure
4D Cap	Semi-Dry	Below 50 KΩ	Silicone Elastomer	Stretchable	0.25–2 N
Chi et al. [35]	Dry	100–500 KΩ	Fabric	not	-
Takumi et al. [26]	Dry	Below 200 KΩ	Straw Hat	not	8–15 N

## Data Availability

The dataset used in this study was not chosen to be publicly available.
